# Interplay between singing and cortical processing of music: a longitudinal study in children with cochlear implants

**DOI:** 10.3389/fpsyg.2014.01389

**Published:** 2014-12-10

**Authors:** Ritva Torppa, Minna Huotilainen, Miika Leminen, Jari Lipsanen, Mari Tervaniemi

**Affiliations:** ^1^Cognitive Brain Research Unit, Cognitive Science, Institute of Behavioural Sciences, University of HelsinkiHelsinki, Finland; ^2^Finnish Centre for Interdisciplinary Music Research, University of HelsinkiHelsinki, Finland; ^3^Brain Work Research Centre, Finnish Institute of Occupational HealthHelsinki, Finland; ^4^MINDLab, Center of Functionally Integrative Neuroscience, Aarhus UniversityAarhus, Denmark; ^5^Institute of Behavioural Sciences, University of HelsinkiHelsinki, Finland

**Keywords:** ERPs (event-related potentials), development, attention, auditory memory, neuroplasticity, singing

## Abstract

Informal music activities such as singing may lead to augmented auditory perception and attention. In order to study the accuracy and development of music-related sound change detection in children with cochlear implants (CIs) and normal hearing (NH) aged 4–13 years, we recorded their auditory event-related potentials twice (at T1 and T2, 14–17 months apart). We compared their MMN (preattentive discrimination) and P3a (attention toward salient sounds) to changes in piano tone pitch, timbre, duration, and gaps. Of particular interest was to determine whether singing can facilitate auditory perception and attention of CI children. It was found that, compared to the NH group, the CI group had smaller and later timbre P3a and later pitch P3a, implying degraded discrimination and attention shift. Duration MMN became larger from T1 to T2 only in the NH group. The development of response patterns for duration and gap changes were not similar in the CI and NH groups. Importantly, CI singers had enhanced or rapidly developing P3a or P3a-like responses over all change types. In contrast, CI non-singers had rapidly enlarging pitch MMN without enlargement of P3a, and their timbre P3a became smaller and later over time. These novel results show interplay between MMN, P3a, brain development, cochlear implantation, and singing. They imply an augmented development of neural networks for attention and more accurate neural discrimination associated with singing. In future studies, differential development of P3a between CI and NH children should be taken into account in comparisons of these groups. Moreover, further studies are needed to assess whether singing enhances auditory perception and attention of children with CIs.

## Introduction

Music gives us pleasure and rewards us (Zatorre and Salimpoor, 2013). It helps us in regulation of emotions (Saarikallio, 2010) and even aids in maintaining the healthy functioning of memory and other cognitive functions in old age (Särkämö et al., 2014). Music listening could also be very important for severely hearing-impaired people using cochlear implants (CIs). Unfortunately, perception of music is difficult for CI-mediated listening (Limb and Roy, 2014). The CI substitutes for the sound reception and analysis functions of the inner ear by directly stimulating the fibers of the auditory nerve via an electrode system. The current CI processing strategies are based mostly on extraction and accurate representation of temporal envelopes of sounds (McDermott, 2004), while the spectral information provided to the auditory system is limited and fine structure of sounds is largely lost (Moore, 2003). Further, the dynamic range of electric hearing is highly limited (Moore, 2003; Limb and Roy, 2014). In consequence, the CI recipients have deficits in the ability to perceive many auditory cues important for music perception, like pitch and melody (adults: Drennan and Rubinstein, 2008; children: Hsiao and Gfeller, 2012), timbre (Gfeller et al., 1998; Galvin et al., 2009; Timm et al., 2012) and loudness (Limb and Roy, 2014). However, their rhythm perception is close to normal (Drennan and Rubinstein, 2008; Limb and Roy, 2014).

The music perception of children with CIs (CI children) is affected not only by the degraded auditory input from the CI, but also by the period of deafness (Moore and Linthicum, 2007) and by cognitive factors (Rocca, 2012; Rochette et al., 2014) where the CI children show large variability (Kronenberger et al., 2014). Deafness during the first years of life may harm the development of attentional functions (Houston et al., 2003; Fagan and Pisoni, 2009) and their neural determinants (Moore and Linthicum, 2007). There is, however, almost a complete lack of studies on CI children's auditory attention even though it is important for their auditory learning (Houston and Bergeson, 2014) and for perception of degraded speech (Wild et al., 2012). Therefore, we investigate how effectively the CI children shift their attention toward changes in the auditory environment. Shifting of attention brings potentially important information into focus, allowing re-evaluation of the entire situation (Horváth et al., 2008).

Longitudinal studies in normal-hearing (NH) adults and children imply a causal link from musical training and hobbies to changes in brain structure and function (plasticity) (Herholz and Zatorre, 2012; Putkinen et al., 2013a, for a review), processing of sound features important for music (Moreno et al., 2009; Chobert et al., 2012) and auditory attention (Fujioka et al., 2006). It has been shown that formal musical training can facilitate the perception of pitch, melodic contour, musical timbre, and general music perception of CI users (Galvin et al., 2009; Yucel et al., 2009; Chen et al., 2010; Hsiao and Gfeller, 2012; Petersen et al., 2012; Limb and Roy, 2014) and it has been suggested that auditory attention of CI children can be facilitated with musical education (Rochette et al., 2014). In addition, informal musical activities at home, including singing, can facilitate the sound discrimination and attention of children (Putkinen et al., 2013a,b). Singing is an essential aspect in the development of musicality and has been recommended to be used in the rehabilitation of music perception of CI children (Rocca, 2012). Importantly, singing also captures and maintains the attention of young normal-hearing children (Nakata and Trehub, 2004) as well as CI children and is therefore effective in their speech therapy sessions (Ronkainen, 2011). Production of songs might play a special role for auditory attention because during singing the child not only hears but also feels the sounds in his/her articulatory apparatus, and multisensory stimuli recruit the attention of the young child (Bahrick and Lickliter, 2000). However, more research is needed on the role of informal singing in CI children's musical and attention development.

Here, we study how the CI children build up their music perception using singing. For our research purposes, we use event related potential (ERP) recordings. Mismatch negativity ERP response (MMN; Näätänen et al., 1978) is a cortical response to a moderate violation of regularity in the auditory scene (Wetzel et al., 2006). In NH listeners MMN has been shown to become larger in amplitude with more accurate perception and with effective discrimination training (Kujala et al., 2007). In the adult CI users, MMN becomes larger with more accurate discrimination of musically relevant sound changes such as pitch, rhythm and timbre (Timm et al., 2014) and increased time of CI use (Lonka et al., 2013). MMN latencies, in turn, are shortened by increasing physical difference between the standard and deviant tone (Kujala et al., 2007). MMN can be followed by a P3a response, which reflects an involuntary attention switch toward a salient sound change in the auditory environment (Escera et al., 1998; Wetzel et al., 2006). Like MMN, P3a for deviant tones becomes larger with increasing physical difference between the deviant and standard stimulus (Winkler et al., 1998; Wetzel et al., 2006) and with effective auditory training (Uther et al., 2006). Further, P3a is larger and earlier in CI children with better speech recognition (Kileny et al., 1997). P3a is thought to reflect evaluative discrimination related to the activation of an attentional switch mechanism (Friedman et al., 2001; Horváth et al., 2008). This is in contrast to the pre-attentive detection of deviant events reflected by the MMN (Tremblay et al., 1998; Friedman et al., 2001; van Zuijen et al., 2006). It seems that the P3a is elicited when the sound change is significant and intrusive for the listener (Horváth et al., 2008).

We measured the MMN and P3a responses to changes in sound pitch, timbre, duration, gap, and intensity in a passive multi-feature paradigm (MFP) (Näätänen et al., 2004; Torppa et al., 2012) using instrumental sounds. We chose to use MFP since it provides a comprehensive view of the basic auditory processing during a single recording in a shorter period of time than with traditional oddball paradigms. This is highly beneficial in child measurements (Lovio et al., 2009), which was important for the present study where the participants were aged from four to thirteen years. There is almost a complete lack of studies on the neural encoding of the sound feature changes in musical instrument stimuli in deaf-born, early-implanted CI children (however, see Torppa et al., 2012) and nothing is known about the development of MMN and P3a to musical instrument sounds in children, within the time period we are concentrating on (14–17 months). The CI children participating the present study, however, were implanted so early (at or before age of 3 years, 1 month) that they had the possibility to develop many auditory functions similar to NH children (Kral and Sharma, 2012). Because typically the amplitude of ERP responses increases and latency decreases as a function of auditory experience with CIs (P1 latency: Kral and Sharma, 2012; MMN amplitude: Ponton et al., 2000; Lonka et al., 2013; P3a amplitude: Kelly et al., 2005), we expect that the MMN and P3a of the present participants with CIs would develop more during our follow-up than those of NH children.

We tested two hypotheses based on the introduction above: (I) The CI children have smaller and/or later MMN and P3a than NH children for the changes in timbre and pitch (while not for the rhythm-related changes i.e., gaps and duration changes): the differences between groups become smaller over time. (II) The MMN and P3a is/becomes larger and/or earlier in CI children who sing regularly at home compared to other CI children. To follow and compare development between groups, the experiment was performed twice. To test Hypothesis II, we divided the CI children into two groups based on the regularity and amount of their long-term singing at home: “CI singers” and “CI non-singers.”

## Materials and methods

### Participants

The CI participants of this study (CI group) were 21 (9 male, 12 female) unilaterally implanted, congenitally deaf, Finnish-speaking children aged from 4 to 13 years during the course of this study (Table 1). They were chosen from a total of 30 children with a CI. Inclusion criteria were switch-on of the CI prior to three years one month, no re-implantation between the two measurements, more than 6 CI channels in use, and the absence of any diagnosed additional developmental or linguistic problems. All CI children had been using their implants continuously for at least 30 months prior to first measurement (T1) and at least 46 months prior to the second measurement (T2), had full insertion of the electrode and attended mainstream school or day care. Seventeen CI children used Nucleus and four used Medel devices (Table 1). According to the clinical recordings, their hearing thresholds in the unimplanted ear were so high that they could not benefit from residual hearing in the present ERP measurements. They all also participated in the study by Torppa et al. (2012) and Torppa et al. (2014), as did the 22 normal-hearing children who served as a control group (NH group).

**Table 1:** **Characteristics of the participants**.

**ID^a^**	**Age at T1**	**Hand^b^**	**Music^c^**	**SE^d^**	**Etiology^e^**	**Age at switch-on of CI (months)**	**CI use prior T1 (months)**	**CI processor type^f^**	**Pure tone thresholds using CI (dB HL)^g^**
CIs 01	5y 11m	R	20(betw)	R	U	18	53	NF	25/22/25/-
CIs 03	9y 2m	R	12(betw)	R	U	32	77	MT	35/28/-/-
CIs 04	7y 10m	R	24(betw)	R	U	25	69	MT	25/23/25/25
CIns 09	7y 4m	R	0(betw)	R	C	19	69	MO	35/35/35/-
CIs 13	5y 5m	R	22(betw)	R	U	18	47	NE	35/32/30/45
CIs 14	4y 4m	R	0(betw)	R	U	18	34	NF	15/17/20/30
CIs 15	5y 1m	R	0	R	C	17	44	NE	45/43/40/40
CIns 16	7y 2m	R	0	R	C	25	61	NF	25/20/35/35
CIns 17	9y 4m	L	0	R	U	19	93	NF	30/25/25/40
CIns 18	12y 1m	R	0	R	U	27	118	NF	25/15/30/45
CIns 19^*^	7y 5m^*^	R^*^	0^*^	R^*^	U^*^	29^*^	60^*^	NE^*^	20/30/35/40
CIs 20	5v 8m	R	0	R	U	20	48	NF	30/27/35/30
CIs 21	5y 7m	L	0	L	C	19	48	NF	25/28/40/40
CIs 22	7y 1m	R	0	R	U	21	48	NE	35/28/35/35
CIns 23	7y 10m	L	0	R	U	18	76	MT	30/25/30/30
CIns 24	4y 2m	R	23(betw)	R	C	14	36	NF	20/20/25/40
CIs 26	4y 2m	R	23(betw)	R	C	20	30	NF	20/23/30/-
CIns 27	4y 2m	R	0	R	C	13	37	NF	25/30/30/-
CIs 28	6y 2m	R	24	R	U	22	52	NF	10/10/10/55
CIns 29	8y 7m	R	0	L	C	37	66	NF	25/28/30/25
CIs 30	6y 7m	R	0	R	C	25	54	NF	40/28/30/-
N CI = 21	Mean = 6y 7m	N R+L = 18+3	N attend:	N R+L = 19+2	N U = 12	Mean = 21.7	Mean = 58.1	N NF = 13	Mean CIs
N CIs = 12			before = 7		N C = 9			N NE = 4	28/26/29/38
N CIns = 9			betw = 8					N MO = 1	Mean CIns
								N MT = 3	26/25/31/36
NH 02	7y 11m	R	36(betw)						
NH 03	4y 6m	R	0						
NH 04	8y 2m	R	45(betw)						
NH 05	10y 0m	R	0(betw)						
NH 06	5y 8m	R	0(betw)						
NH 07	6y 9m	R	0						
NH 08	5y 7m	R	0(betw)						
NH 09	4y 6m	L	42(betw)						
NH 10	4y 0m	R	0(betw)						
NH 11	5y 6m	R	0						
NH 13	5y 0m	R	35(betw)						
NH 14	4y 6m	R	15(betw)						
NH 15	12y 0m	R	0						
NH 16	8y 5m	R	0						
NH 17	9y 8m	R	0						
NH 18	6y 9m	R	0						
NH 19	7y 0m	R	0						
NH 20	4y 6m	R	12						
NH 21	6y 5m	R	15						
NH 22	6y 11m	R	0(betw)						
NH 23	5y 5m	R	12						
NH 30	11y 2m	L	54(betw)						
N NH = 22	Mean = 6y	N R + L = 20+2	N attend						
	9m		before = 9						
			betw = 11						

CI, child using cochlear implant; NH, normal-hearing child; CIs, CI child who sang regularly at home; CIns, CI child who did not sing regularly; T1, first time point of measurements; T2, second time point of measurements; N, number.

* Included in analyses but data only from T2.

a Identification number.

b Hand, handedness.

c Music, amount of attending to musical hobbies outside of the home before T1 in months (betw) = child attended musical hobbies outside of the home between measurements (dancing excluded).

d SE, stimulated ear.

e U, unknown, C, Connexin 26.

f NF, Nucleus Freedom, implant type CIC4 (coding strategy: ACE).

NE, Nucleus ESPrit 3G, implant type CIC3 (coding strategy: ACE).

MT, Medel Tempo + (coding strategy: CIS).

MO, Medel Opus 2 (coding strategy: CIS).

g For 4000 Hz / for mean of 500, 1000, and 2000 Hz / for 250 Hz / for 125 Hz.

The 22 NH children (11 male, 11 female) were siblings of the participating CI children or were invited for this study from the neighborhood of the first author, from local musical play schools, and from another ongoing study at the University of Helsinki. The NH group was matched to the CI group in group level as accurately as possible by age, gender, handedness as well as social and musical background, the latter as assessed by attendance in musical playschool or related musical activities (see Table 1). None of the NH children had any diagnosed developmental or linguistic problems. Their hearing was found to be normal in regular check-ups at child welfare clinics.

Parents of the participants gave a written informed consent and the participants gave their consent verbally. The study was carried out in accordance with the Declaration of Helsinki and all procedures were approved by the ethical committees of the participating hospitals.

#### Division of CI group into CI singers and CI non-singers

We made the division with similar principles as in the previous child studies comparing children with and without musical training (schools with emphasis on music, Putkinen et al., 2014; private instrument lessons, Strait et al., 2012; see Margulis, 2008, for different criteria for adult musicians vs. non-musicians). Therefore, we chose the CI singers on the basis of the regularity of their musical activity (singing) in the home and the time they had sung before the study began. For the division, information about the children's musical and other hobbies as well as musical activities at home, school and day care was collected with questionnaires addressed to parents and personnel at schools or day care. As a measure to divide the CI children into those who sing regularly at home and those who do not, the parents were asked to account for their children's singing activities including those with parents, siblings or alone: “Has your child sung at home? Every week_ every other week_ occasionally_ not at all_ if weekly, how many times in a week” at T1: during the previous year, at T2: between the measurements. According to their answers, 12 CI children sang weekly at home one year before the study began and between T1 and T2 and these are hence called “*CI singers.*” They sang at home on average five times per week before T1 at and 4 times per week between T1 and T2. Nine CI children sang less than weekly or not at all and are called “*CI non-singers*.” They sang at home on average less often than every other week (=occasionally) before T1 and between T1 and T2. In addition, all CI children had at school or day care music lessons with an emphasis on singing.

To ensure that the parents were able to identify singing of the CI children as different from speech, we recorded their singing (“Twinkle twinkle little star”) when they came to the measurements at T2. The parents and children were told about the task beforehand. Nineteen out of 21 CI children completed the task. The rhythm, melody and words (lyrics) they sang were scored blindly, i.e., without knowing whether the child was CI singer or not, by a teacher of singing. As a result, 15 CI children could sing the lyrics correctly and 4 of them partially correctly. Nine of them could sing the rhythm correctly and 6 partially correctly. One CI child sang the melody almost correctly, 5 could vary their pitch partially correctly, 7 varied the pitch to some extent: 6 sang without variation in pitch, however, all of them could produce the rhythm and lyrics correctly or partially. It was concluded that their singing was recognizable and different from general speech. The interviews of parents also supported this conclusion.

The comparisons between CI singers and CI non-singers showed that the accuracy of production of lyrics, melody, and rhythm was better in CI singers than in CI non-singers. Age-controlled ANOVA confirmed that the CI singers were significantly better in production of rhythm [*F*_(1, 18)_ = 7.83, *p* = 0.013] and in the overall accuracy of singing (the mean of production of lyrics, melody and rhythm) [*F*_(1, 18)_ = 5.28, *p* = 0.035] than CI non-singers. In contrast, statistical analyses confirmed that these CI singing groups did not differ from each other in the other home-related musical background (including musical instrument playing), amount of musical activities at day care or schools, in musical hobbies, or in the aspects related to their hearing or CI devices, age, gender, socioeconomic background, or etiology (see Supplement 1).

### Stimuli and procedure

**Figure 1:**
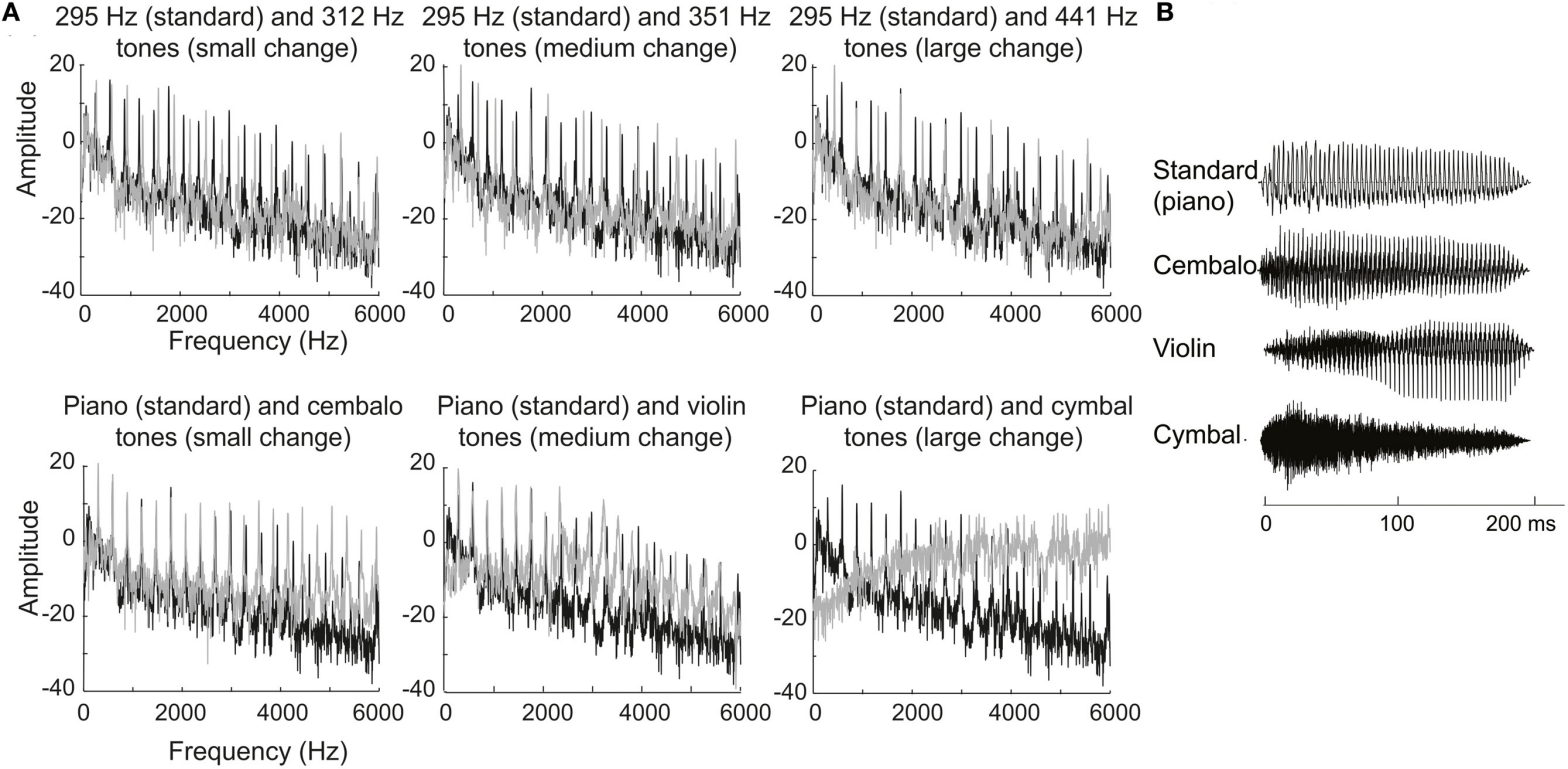
**(A)** Frequency spectra of the standard tone (black) in comparison to pitch and musical instrument deviants (gray) (from Torppa et al., 2012). **(B)** Sound envelopes of the standard piano tone and the musical instrument deviants (from Torppa et al., 2012). The Figures have been reprinted with the permission from Elsevier.

The stimuli and procedure were the same as in Torppa et al. (2012). Piano, harpsichord (cembalo), violin, and cymbal sounds selected from McGill University Master Samples DVD were cut from the beginning of the original samples to the desired duration and normalized in intensity with Adobe Audition 2.0 (Adobe Systems Inc., San Jose, USA). The standard was a piano tone at 295 Hz, duration 200 ms including a 20 ms fall time. The deviant tones differed from the standards at three different levels of fundamental frequency (pitch), all harmonics changing from 295 Hz standard to 312, 351, and 441 Hz corresponding to 1, 3, and 7 semitones respectively, timbre (change from standard piano tone to cembalo, violin, and cymbal tone) (see Figure 1), duration (shortening of 200 ms standard to 175 ms, 100 and 50 ms), intensity (increments and decrements, ±3, ±6, and ±9 dB) or by the presence of a silent gap in the middle of the tone (5, 40, and 100 ms gaps). The deviant tones were equivalent to the standard in all other features, except in the 50 ms deviant, where the fall time was 10 ms, and for the timbre changes, which contained changes in temporal intensity envelope, spectral envelope, and periodicity. In the stimulus sequence standard and deviant tones alternated. The SOA was kept at 480 ms. The presentation order of the changes was randomized throughout the experiment. The probability of the standard tone was 0.5 and the probability of each change was 0.028. There were 2250 standard tones and 125 deviants. The total recording time of the experiment was 36 min.

All stimuli were presented in an acoustically insulated and dampened room through 2 loudspeakers (OWI-202; OWI Inc. CA., USA) placed at a 45° angle to each side of the subject, approximately 1 m in distance from the subject's ear. The stimuli were presented at a fixed and comfortable level, which was 60 dB SPL for NH group and 70 dB SPL for CI group (in exception to the intensity changes). For one CI child the sound level had to be lowered to 65 dB SPL at T1. The sounds were presented using the everyday settings of the CI. Program, and volume and sensitivity level were adjusted to the clinically recommended values. During the experiment, subjects watched a silent video.

### EEG recording, data preprocessing and data analysis

EEG recordings were conducted from a 64-channel electrode cap using Biosemi ActiveTwo amplifier and active electrodes (Biosemi B.V., Netherlands) (sampling rate of 512 Hz, band-pass filtering of DC-102.4 Hz). The data were referenced on-line to the CMS electrode according to the basic setup of the Biosemi measurement device and off-line data was re-referenced to the electrode placed at the nose tip. Additional electrodes were placed at the left and right mastoid and to record eye movements and blinks. EEG data were analyzed using EEGLAB 8 (Delorme and Makeig, 2004). Imported data were downsampled at 256 Hz, and high-pass filtered above 0.5 Hz. Because of the location of the CI device, some channels could not be used; data from these electrodes were interpolated. The analysis epoch was 550 ms long, starting 100 ms before the onset of the tones. The baseline level of the epochs was set to be zero during the 100 ms before the tone onsets.

Independent component analysis (ICA) with the Fastica algorithm was applied to remove ocular and muscle artifacts in both CI and NH groups (Makeig et al., 2004). In addition, ICA was used in the CI group to reduce the CI related artifact. Several previous studies indicate that ICA is the best available method for this (Gilley et al., 2006; Sandmann et al., 2010: for the CI artifact component, see Torppa et al., 2012). Before ICA, data dimensionality was narrowed down by the amount of interpolated channels, and automatic epoch rejection at a threshold between ±300 and ±400 μV was performed. The rejection thresholds were individually adjusted to preserve at least 85% of original epochs for effective statistical analysis. After ICA, the epoch voltage rejection was done again with a threshold of ±150 μV. Further, the proportion of remaining epochs after voltage rejection was analyzed for each individual subject. The criteria of 75% (95) remaining epochs for each deviant was used. One participant did not reach the criteria at T1 and so her data was excluded from the analyses. All participants reached the criteria at T2 and thus the data from all children at T2 was used in further analyses. The mean percentage of acceptance of epochs at T1 was 94% in the CI group (119 deviants, 2348 standards) and 93% in the NH group (116 deviants, 2330 standards), and at T2 was 93% in the CI group (116 deviants, 2330 standards) and 95% in the NH group (119 deviants, 2348 standards). To increase the signal to noise ratio (especially for noise sensitive latency measures), and to make the analyses more comparable between groups, F3, Fz, F4, C3, Cz, and C4 channels were averaged to form a ROI (region of interest) channel. This procedure was used in all ERP analyses.

Calculating the median instead of average of ERP signals is optimal in cases where the data are of high quality in general, but some overlapping noise seen as extreme values is expected, when studying young children (Yabe et al., 1993). Hence we chose to use this median method in the present study (for differences between median and averaged signals, see Supplements 2, 3). First, the trials of each individual were grouped by stimulus type. After this, the median value of the signal amplitude values of one sample point was taken as representative of that sample point. Thus, the resulting curve from an individual consists of the samples having the median amplitude over the accepted trials. Further, the data were offline-filtered with a 25 Hz low-pass filter and the baseline level of the epochs was set to be zero during the 50 ms period before the tone onsets.

MMN was identified as the local minimum (most negative peak) of the subtraction waveform within the time window 90–250 ms after change onset. P3a was identified as the local maximum of the subtraction waveform within the time window 145–300 ms after change onset. Gap MMN and P3a was defined in relation to offset of the gap due to the clear tendency of the CI group to have MMN only for the offset of 100 ms gap. The mean amplitudes were calculated using a 30 ms time window surrounding the peak latency. The individual peak latencies were calculated from the ROI-signal in a time window determined in relation to the onset of the stimulus change. For gap changes, the time window was determined in relation to the offset of the gap, and for duration changes, in relation to offset of the deviant tone. Based on visual inspection of the data, the window was set at 85–250 ms for pitch and timbre MMN, at 100–250 ms for duration and gap MMN and at 145–400 ms for P3a. The onset of the timbre and pitch MMN latency window was early, since the earliest individual MMN for which we could confirm polarity change at mastoid electrodes compared to Fz was found for timbre changes at 86 ms and for pitch changes at 89 ms. In addition, we analyzed the amplitudes and latencies of the intensity decrement and increment MMN and P3a responses (analyses and results are provided in Supplement 4).

ERP response amplitudes and latencies were subjected to one-sample, two-tailed *t*-tests in order to examine whether they significantly differed from zero in the CI and NH group. In order to compare MMN and P3a between CI and NH groups or between CI singers and CI non-singers, we took into analysis the responses with following principles. The response for the specific deviant type was included in the analyses, if the MMN/P3a was significant at T1 and/or T2 for the both tested child groups. Cases, where the significant response was not found either at T1 or T2, were still included in the analyses, because the standard deviation of the responses were similar in both measurement points (see Table 2).

**Table 2:**
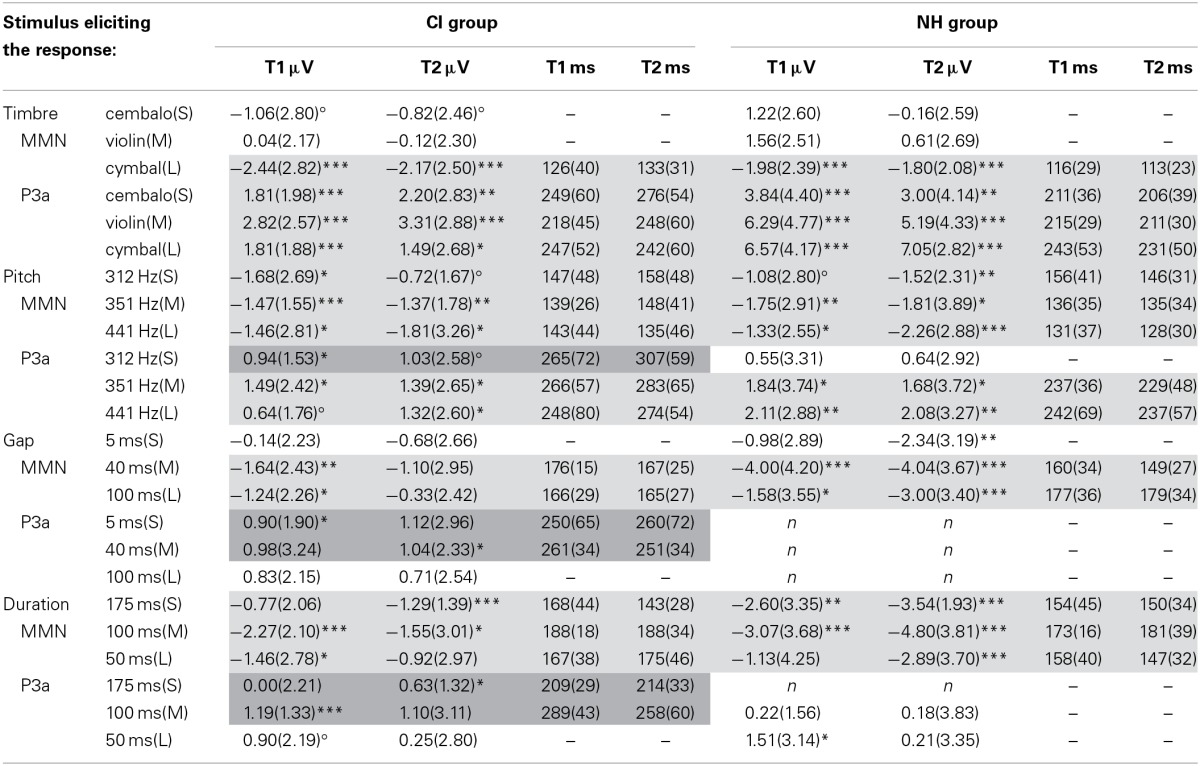
**The MMN and P3a mean amplitudes and latencies**.

For both CI and NH groups, the mean MMN and P3a amplitude (standard deviation in parenthesis), followed by the significance of the response (°p < 0.1, ^*^p < 0.05, ^**^p ≤ 0.01, ^***^p < 0.001; two-tailed t-test against zero), are given at T1 and T2. These are followed by the mean of peak latencies (standard deviation in parenthesis). Light gray presents the amplitude and latency values included in statistical comparisons between CI group and NH group as well as between CI singers and CI non-singers. Dark gray presents the values included only in statistical comparisons between CI singers and CI non-singers. – = the individual latencies were not analyzed. n = the responses were non-existent (wrong polarity in the time window of the response). Standard was 295 Hz, 200 ms, 70 db (for CI group)/60 dB (for NH group) piano tone. S, M, L = small, medium and large amount of change.

### Statistical analyses

The analyses for testing the hypotheses were conducted with Linear Mixed Model (LMM; Singer and Wilett, 2003; West, 2009). This multilevel statistical procedure enables studying individual change over time to fit a variety of advanced regression models to longitudinal data sets. LMM was used because (a) it takes into account the missing values, which makes it possible to utilize data from all participants even though the data at one of the two time points are missing (as mentioned before, we had to exclude data from one CI participant at T1), (b) LMM allows one to combine measurements from the two time points in a single analysis, which allowed us to compare the development of the ERP indices of auditory perception and attention over time in CI and NH participants.

Two LM regression analysis models were created to test the hypotheses I and II. According to the hypotheses, the first LMM included factor CI vs. NH group (*group*) and the second LMM included factor CI singers vs. CI non-singers (*CI singing group*). In both LMMs, the amplitude or latency values from recording times T1 and T2 were included (factor *time*). Accordingly, the interactions of *time* with *group* or *CI singing group* were tested to determine differences between child groups in development. In both LM models, *amount* of change was also a factor, and two- and three-way interactions of that with *group* or *CI singing group* and *time* were tested. Two-way interactions were tested to find out whether there would be differences between aforementioned groups only in some particular amount of change, and three-way interactions were tested to find out whether there would be differences in the development between any of the groups only in some particular amount of change. We included *age* as a covariate in all analyses to control for the large age range of the participants. Interactions with *age* were also tested because interaction of *age* with *group, CI singing group* or *time* would indicate that age could not be controlled. Only the significant interactions of *age* are reported in Tables 3, 4. All non-significant interactions were omitted from the final results reported in Tables 3, 4. Because the amounts of changes across the change types were not matched, the hypotheses had to be tested separately for each change type.

**Table 3:** **Results for testing Hypothesis I**.

	**Timbre MMN (L)**		**Timbre P3a (S, M, L)**				**Pitch P3a (M, L)**		**Gap MMN (M, L)**				**Duration MMN (S, M, L)**			
	**Latencies**		**Amplitudes**		**Latencies**		**Latencies**		**Amplitudes**		**Latencies**		**Amplitudes**		**Latencies**	
	***B***	***F***	***B***	***F***	***B***	***F***	***B***	***F***	***B***	***F***	***B***	***F***	***B***	***F***	***B***	***F***
Group	−14.82	4.92^*^	5.14	17.42^***^	−19.25	8.30^**^	−32.01	6.12^*^	−2.10	8.74^**^	12.61	0.16	−2.49	6.66^*^	−10.61	4.87^*^
Time	−1.20	0.03	0.18	0.28	−15.50	0.69	−6.09	0.55	0.04	0.01	4.71	1.25	2.40	10.10^**^	3.71	0.74
Amount		–	−1.18^a^	7.87^***^	18.20^a^	0.014^*^	3.21^b^	0.70	−1.18^b^	8.68^**^	40.43^b^	3.34°	−0.48^a^	6.70^a^^**^	−8.03^a^	15.80^***^
			3.64^b^		−11.15^b^								−1.35^b^		20.69^b^	
Time × group		ns		ns		4.99^*^		ns		ns		ns		7.65^**^		ns
Amount × group		–		10.72^***^		7.81^***^		ns		ns		12.09^***^		ns		ns

Group = CI vs. NH group. Amount = amount of change. Following the response type, in parentheses the amount of changes included in analysis: S, M, L = small, medium, large amount of change. B shows the direction of the connection. For B: group: reference is the CI group. Time: reference is the T2.

a B for small change, reference is the large change.

b B for medium change, reference is the large change. – = interaction was not included in analysis. ns = interaction was included in analysis: that was omitted from final results because that was not significant. Age was always controlled.

° p < 0.1,

* *p* < 0.05,

** *p* = 0.01,

*** *p* ≤ 0.001; two-tailed t-test against zero.

**Table 4:** **Results for testing Hypothesis II**.

	**Timbre MMN (L)**		**Timbre P3a (S,M,L)**				**Pitch MMN (S, M, L)**		**Pitch P3a (S, M, L)**				**Duration MMN (S, M, L)**	
	**Amplitudes**		**Amplitudes**		**Latencies**		**Amplitudes**		**Amplitudes**		**Latencies**		**Amplitudes**	
	***B***	***F***	***B***	***F***	***B***	***F***	***B***	***F***	***B***	***F***	***B***	***F***	***B***	***F***
Group	4.07	0.19	−2.71	4.19°	64.98	7.07^*^	−4.12	3.52°	−1.52	5.36^*^	36.30	7.14^*^	−1.76	1.49
Time	11.21	10.82^**^	−1.02	0.01	3.69	0.62^*^	−1.28	0.12	−0.22	0.40	−27.05	5.83^*^	2.38	5.60^*^
Amount		–	0.36^a^	7.17^***^	18.20^a^	5.04^**^	−0.42^a^	1.02	−0.01^a^	0.78	25.15^a^	1.69	0.14^a^	2.28
			1.43^b^		−11.15^b^		−0.61^b^		0.45^b^		13.62^b^		−0.72^b^	
Time × group		13.21^**^		10.15^**^		8.81^**^		5.40^*^		ns		ns		4.46^*^
Time × group × amount		–		ns		ns		2.42^*^		ns		ns		ns
Time × age × group		9.80^***^		ns		ns		ns		ns		ns		ns

Group = CI singing group, i.e., CI singers vs. CI non-singers. Amount = amount of change. Following the response type, in parentheses the amount of changes included in analysis: S, M, L = small, medium, large amount of change. B shows the direction of the connection. For B: Group: reference is the CI singers. Time: reference is the T2.

a B for small change, reference is the large change.

b B for medium change, reference is the large change. – = amount or interaction was not included in analysis. ns = interaction was included in analysis: that was omitted from final results because that was not significant. Results for age are given only when that could not be controlled.

° p < 0.1,

* *p* < 0.05,

** *p* ≤ 0.01,

*** *p* ≤ 0.001, two-tailed t-test against zero.

Importantly, in multilevel analyses, the covariance structure must always be taken into account. LMM procedure allows testing and usage of the best fitted covariance structure. Therefore, a statistical correction like Greenhouse-Geisser is not needed. We tested the covariance structures and selected the best fitting ones based on Akaike's and Bayesian information criteria (AIC and BIC) (Bryk and Raudenbush, 2002). AIC is a measure of the relative quality of a statistical model for a given set of data (Akaike, 1974), while BIC is a criterion for model selection among a finite set of models (Schwarz, 1978). The results are reported with the best fitting structure. The Bonferroni correction was used for all *post-hoc* tests within each model and for those tests, only the Bonferroni corrected *p* values are given.

In Torppa et al. (2012), small or non-existent MMN preceding early P3a for changes in timbre was found. This suggested that the small MMN was consequence of the partial overlap of the P3a with the MMN, i.e., that the P3a was elicited early, at the latency where the MMN was assumed to be elicited. If in the present study the MMN preceding the P3a was unexpectedly small, we conducted partial correlation analysis (age controlled) between the amplitudes of the MMN, or the ERP-responses in the expected time line of the MMN, and the amplitudes of the following P3a. If the correlation was positive, the MMN became smaller with enlargement of the P3a, and the overlap was evident.

In general, the critical level for significance was set at 0.05, and only the significant results related to the hypotheses are reported in the Results Section. All statistical analyses were made with SPSS 20 software.

## Results

Table 2 shows the mean amplitudes and the latencies of the MMN and P3a responses for the timbre, pitch, gap, and duration deviants for the CI and NH groups. Results related to intensity increments and decrements are provided in Supplement 4.

### Timbre and pitch responses were small and/or late in the CI group

**Figure 2:**
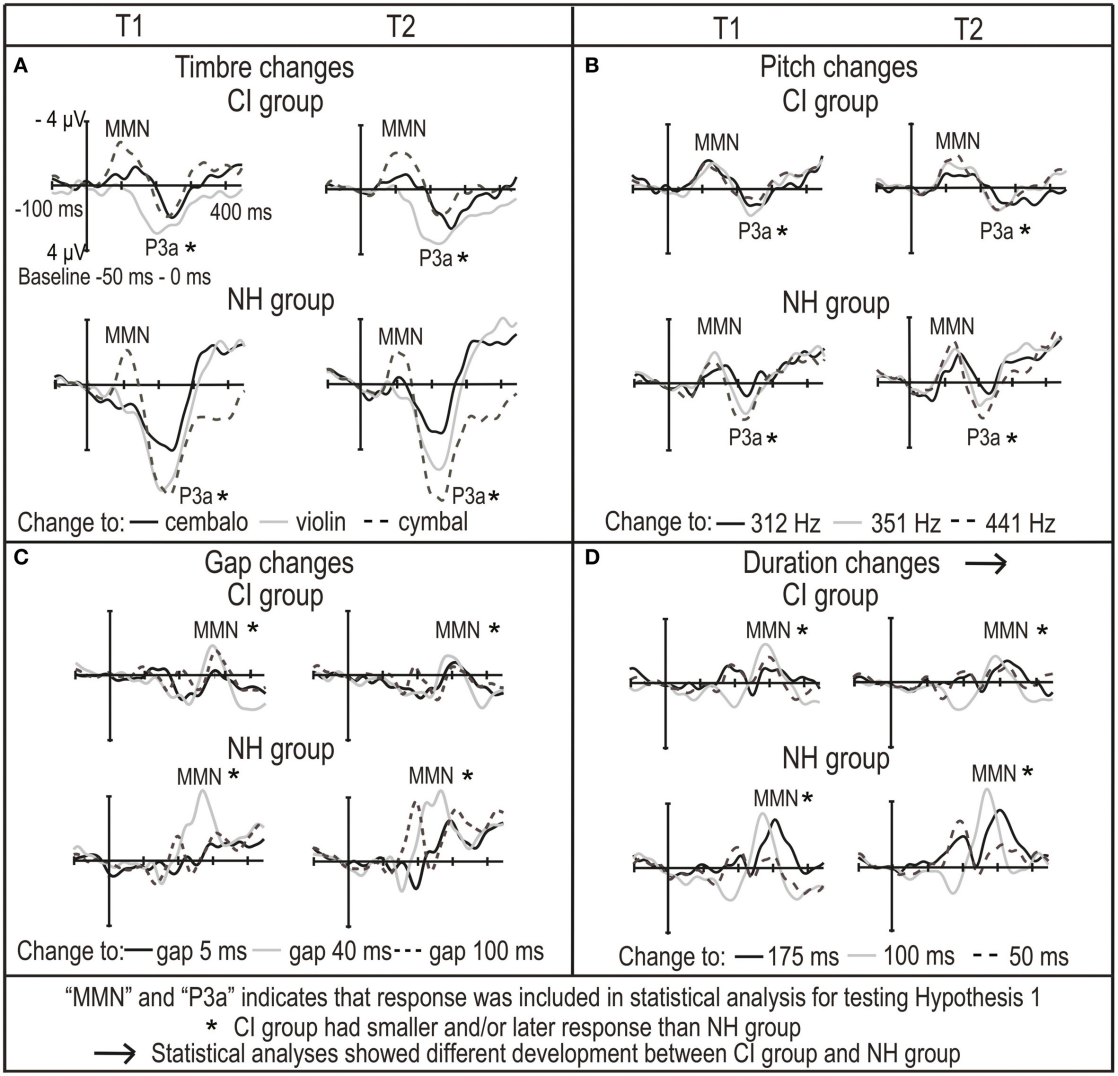
**The subtraction (deviant—standard) ROI waveforms averaged across F3, Fz, F4, C3, Cz and C4 electrodes for CI and NH group for (A) timbre changes, (B) pitch changes, (C) gap changes and (D) duration changes**. They are given for both time points of the measurements (T1 and T2 on the left and right in each panel, respectively).

Timbre MMN latency was longer and timbre P3a was smaller and later in the CI group than in the NH group (main effects of *group*, Table 3; Figure 2A, for ERP waveforms, Supplement 5). Because there was a significant interaction of *group* and *amount* of change for both P3a amplitude and latency (Table 3), we conducted *post-hoc* tests. We found that the difference between the groups was significant for each amount of timbre change (medium change, i.e., from piano to violin: amplitude, *p* = 0.003; large change, i.e., to cymbal, amplitude, *p* < 0.001; small change, i.e., to cembalo, latency, *p* < 0.001). In addition, timbre P3a latency became longer over time only in the CI group (interaction of *group* and *time*, Table 3; *post-hoc* test, *p* = 0.035).

**Figure 3:**
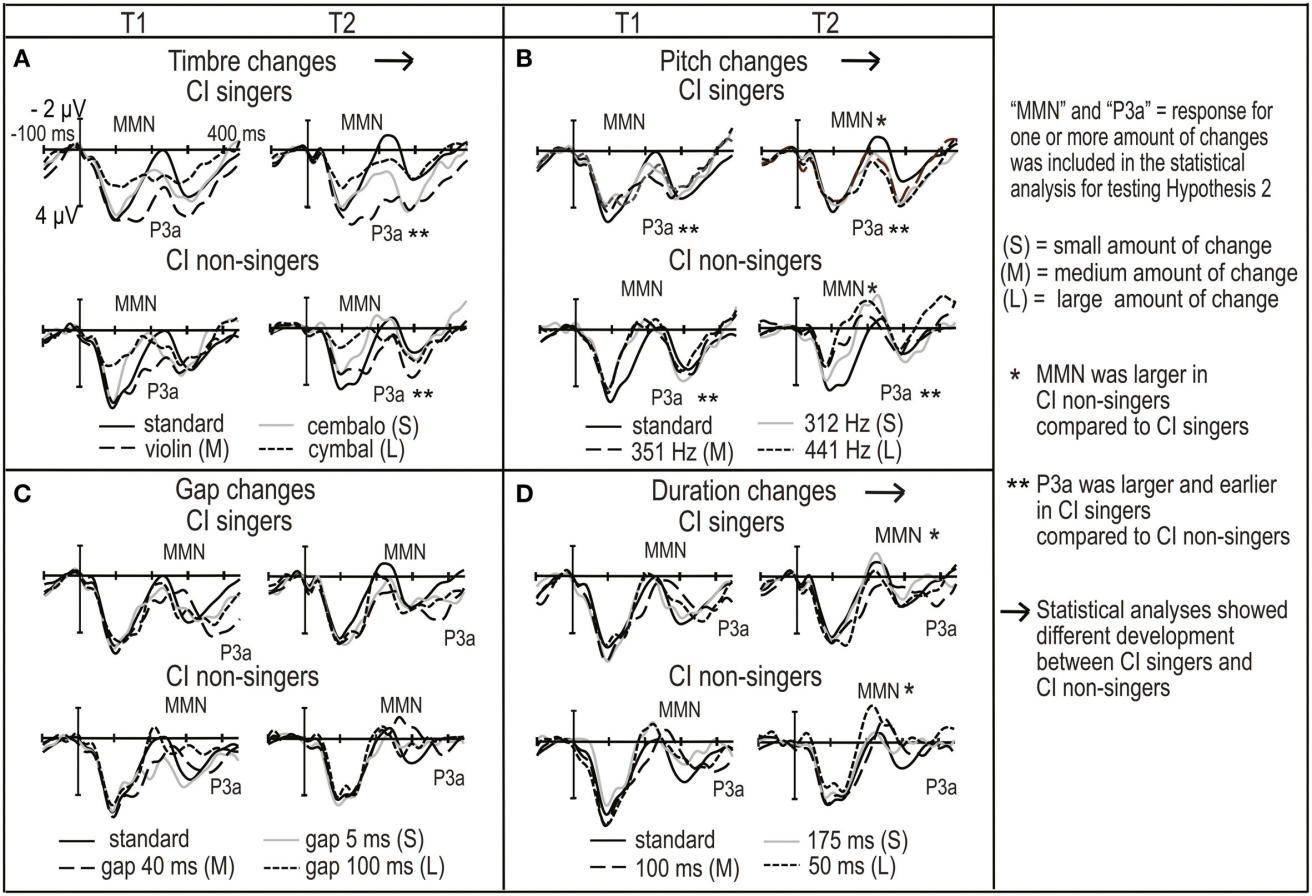
**The ERP ROI waveforms averaged across F3, Fz, F4, C3, Cz, and C4 electrodes for CI singers and CI non-singers for standard tones and for (A) timbre changes, (B) pitch changes, (C) gap changes and (D) duration changes**. The ERP waveforms are given for both time points of the measurements (T1 and T2 on the left and right in each panel, respectively).

**Figure 4:**
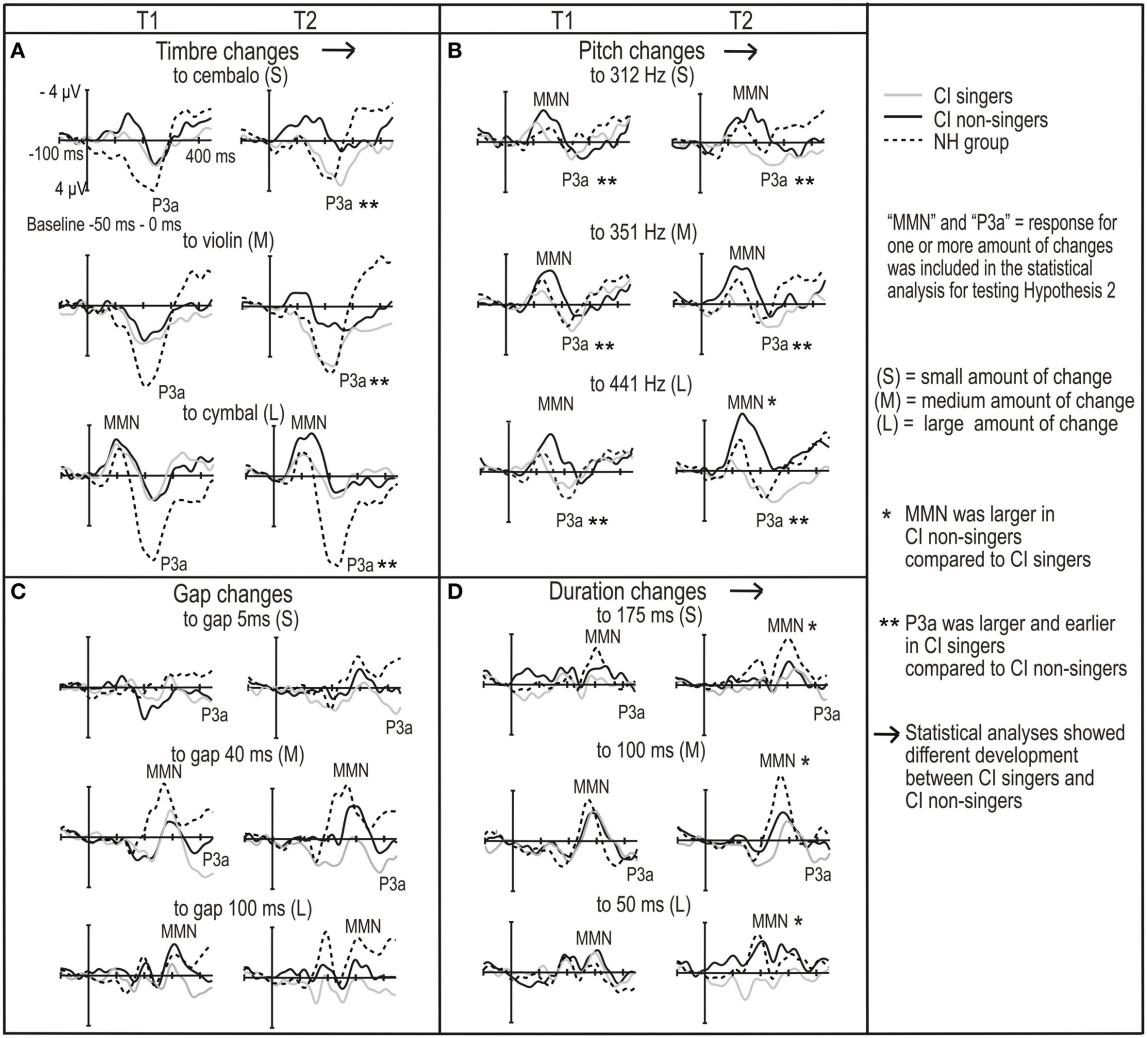
**The subtraction (deviant—standard) ROI waveforms averaged across F3, Fz, F4, C3, Cz and C4 electrodes for NH group, CI singers and CI non-singers for **(A)** timbre changes, **(B)** pitch changes, **(C)** gap changes and **(D)** duration changes**. The ERP waveforms are given for both time points of the measurements (T1 and T2 on the left and right in each panel, respectively).

Moreover, in the NH group, the MMN was non-existent for the change from piano to cembalo and to violin (Figure 2A). This was also the case in the CI singers (Figures 3A, 4A). Therefore, we conducted the partial correlation analyses for all participants between the amplitudes the timbre MMN (at the latency it was assumed to be elicited) and the timbre P3a. We found that MMN and P3a amplitudes for the changes to cembalo and to violin correlated significantly (at T1, cembalo, *r*_*p*_ = 0.48, *p* = 0.001; violin, *r*_*p*_ = 0.65, *p* < 0.001; at T2 violin, *r*_*p*_ = 0.49, *p* = 0.001): the MMN was smaller when the P3a was larger. This correlation suggests a co-dependence and a possibly overlapping MMN and P3a (see Discussion).

Pitch P3a latency was longer in the CI group than in the NH group (main effect of *group*, Table 3, Figure 2B). Gap MMN amplitude was smaller and latency for the medium gap longer in the CI group compared to the NH group (amplitude, main effect of *group*, Table 3; latency, interaction of *amount* of change and *group*, Table 3; *post-hoc* test, difference between CI and NH group for the medium change, *p* = 0.013) (Figure 2C, Supplement 5). Importantly, gap P3a was elicited in the CI group only (Figure 2C). We also conducted partial correlation analyses between the amplitudes of the MMN and P3a in the CI group. They correlated significantly (small gap, at T1, *r*_*p*_ = 0.59, *p* = 0.008, at T2, *r*_*p*_ = 0.67, *p* = 0.001; medium gap, at T1, *r*_*p*_ = 0.54, *p* = 0.018, at T2, *r*_*p*_ = 0.55, *p* = 0.012; large gap, at T2, *r*_*p*_ = 0.58, *p* = 0.007). The MMN was smaller with the larger P3a, suggesting overlapping gap MMN and P3a in the CI group (see Discussion).

Duration MMN amplitude was smaller and latency longer in the CI group than in the NH group (main effects of *group*, Table 3; Figure 2D). Duration MMN became larger by time only in the NH group and was at T2 significantly smaller in the CI group than in the NH group (interaction of *group* and *time*, Table 3; enlargement over time in the NH group, *p* = 0.001; difference between groups at T2, *p* = 0.001). Importantly, at T1, the duration MMN was followed by P3a in both groups. In contrast, at T2, the P3a was elicited only in the CI group (Table 2, Figure 2D). Therefore, we conducted the partial correlation analyses between the MMN and P3a amplitudes at T2 for the CI group. They correlated significantly (small change, *r*_*p*_ = 0.64, *p* = 0.001; medium change, *r*_*p*_ = 0.68, *p* = 0.001; large change, *r*_*p*_ = 0.79, *p* < 0.001). The MMN was smaller when the P3a was larger, suggesting a possible overlap of MMN and P3a for the duration deviants in the CI group (see Discussion).

### P3a development was enhanced in the CI singers

**Figure 5:**
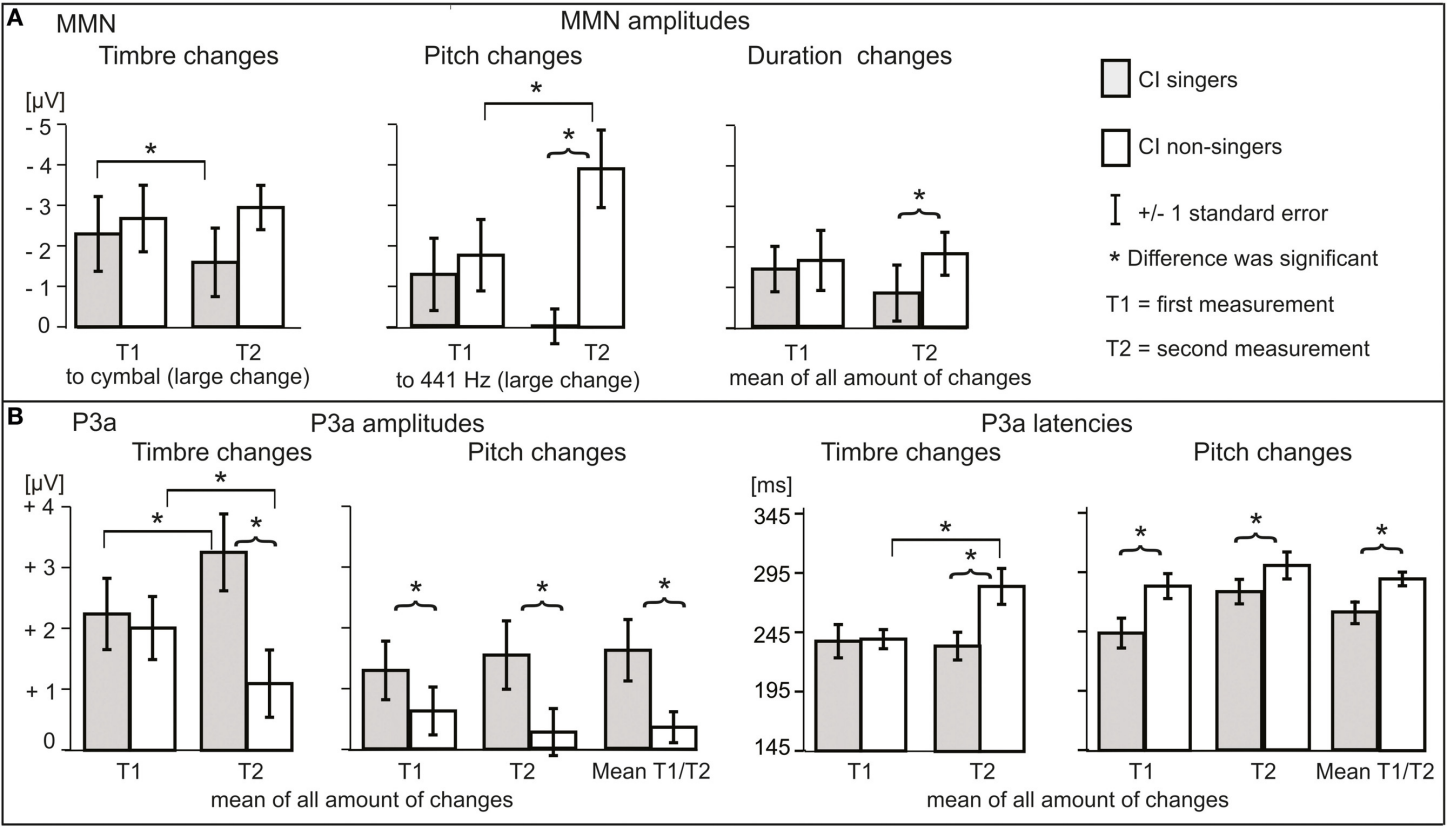
**The illustration of significant differences between CI singers and CI non-singers in (A) MMN and (B) P3a responses**.

Timbre MMN amplitude became smaller over time only in the CI singers (interaction of *time* and *group*, Table 4; *post-hoc* test, *p* = 0.005) (Figures 3A, 4A, 5A). The interaction of *time*, *age*, and *group* was significant (Table 4).

Timbre P3a was earlier in the CI singers than in the CI non-singers (main effect of *group* for latency, Table 4). Timbre P3a became larger over time in the CI singers but smaller and later over time in the CI non-singers and was significantly smaller and later in CI non-singers than in the CI singers at T2 (for the interactions of *time* and *group*, Table 4) (*post hoc* tests: in the CI singers, P3a amplitude increases, *p* = 0.017; in the CI non-singers, P3a amplitude decreases, *p* = 0.036, P3a latency becomes longer, *p* = 0.001; P3a amplitude difference between groups at T2, *p* = 0.005, P3a latency difference between groups at T2, *p* = 0.001) (Figures 3A, 4A, 5B).

Pitch MMN amplitude was larger in the CI non-singers than in the CI singers (main effect of *group*, Table 4). In the CI non-singers alone, pitch MMN amplitude became larger over time especially for the large change, and was at T2 significantly larger in them than in the CI singers (interaction of *time* and *group*, as well as *time, amount* and *group*, Table 4: *post-hoc* tests, enlargement of the MMN for the large pitch change, *p* = 0.004; MMN amplitude difference between CI singers and CI non-singers for the large change at T2, *p* < 0.001) (Figures 3B, 4B, 5A). The pitch P3a of the CI non-singers, however, did not become larger over time with the pitch MMN. Importantly, the pitch P3a was larger and earlier in the CI singers than in the CI non-singers (main effects of *group*, Table 4, Figures 3B, 4B, 5B).

The differences between CI singers and CI non-singers in the MMN and P3a for gaps were statistically not significant. However, the difference waves for medium and large gaps were completely positive at T2 in the CI singers but not in the CI non-singers (Figures 3C, 4C).

The CI singers had at T2 significantly smaller duration MMN than the CI non-singers (interaction of *time* and *group*, Table 4; *post-hoc* test, *p* = 0.049) (Figures 3D, 4D and 5A). However, at T2 in the CI singers the difference wave was already positive in the time line of MMN especially for the large duration change (Figure 4D).

## Discussion

We compared CI children and normal-hearing (NH) children as well as the CI singers and the CI non-singers in their development of preattentive discrimination and attention shift toward changes in timbre, pitch, duration, and presence of temporal gaps in musical piano tones. The 4–13 years old children participated in ERP recordings twice (at T1 and T2), in a period between 14 and 17 months. For the first time, we analyzed P3a responses for all change types. We found well-formed MMN and P3a in the CI group resembling those recorded in the NH group, which is in line with the previous findings on CI listeners (Kileny et al., 1997; Koelsch et al., 2004; Torppa et al., 2012). The CI and NH group as well as the CI singers and the CI non-singers differed in MMN and P3a development. Importantly, the P3a development was enhanced in the CI singers.

### The differences between CI and NH group

As expected, timbre P3a was smaller in the CI group than in the NH group. This is in line with previous findings, showing difficulties in discrimination of timbre in CI recipients (see Introduction), and with results on degraded P3a in adult CI listeners (Koelsch et al., 2004; Nager et al., 2007). The present results on non-existent differences in pitch MMN and pitch P3a amplitudes between the CI and NH groups are also in line with Torppa et al. (2012). However, in the present study, we also found that the pitch P3a was later in the CI group. This indicates difficulties in discrimination of piano tone pitch in CI children, which is consistent with previous studies in CI recipients (see Introduction).

Intriguingly, the CI and NH groups differed in the development of MMN and P3a for rhythmic changes (for gaps and the changes in duration), which affects the interpretation of the results on MMN as follows. First, when the MMN for particular rhythmic change (gap, duration) was smaller in the CI group than in the NH group, then the P3a for the rhythmic change was elicited only in the CI group. We also found that in the CI group, the P3a for these rhythmic changes overlapped with the MMN for the corresponding changes, i.e., the P3a peaked already at the assumed latency of the MMN (in the CI singers, see Section Differences Between CI Singers and CI Non-singers). Therefore, because the P3a of the CI group diminished their MMN, the CI vs. NH group comparisons on MMN for rhythmic changes were of little value. Second, in the NH group, when their duration P3a was very small or non-existent, their duration MMN was large. This indicates that the changes in duration became less distractive for the NH group over time (see Wetzel et al., 2006 for the development of distraction and P3a over age in other context), and the lack of the overlap of P3a and MMN allowed their MMN to continue on growing as opposite to the CI group. This is the first time when both the MMN and P3a responses for gaps and duration changes in musical context are compared between CI and NH children. The important message for future research is that these child groups may develop differently on P3a, and this can affect the group comparisons on MMN.

It should be also noted that because the timbre and pitch changes at T2 still elicited P3a in the NH group, their P3a for these changes in musical context may diminish later than P3a for rhythmic changes. Moreover, the rapid development of duration MMN in the NH group could be a consequence of their development in the discrimination of musically relevant duration changes due to their exposure to music inside and outside of the home.

### Timbre P3a without MMN

We found timbre P3a without clear MMN for changes from piano to cembalo and violin. This was evident in the NH group and in part of the CI group, in the CI singers (Figure 4A). P3a without MMN has been found in many previous studies (Wetzel et al., 2006; Horváth et al., 2008; Koistinen et al., 2011, among others), however there seems to be no consensus on their interpretation. We showed for the first time that when the MMN was small or not visible in the subtraction curve, the amplitudes became more positive in the presumed time window of the MMN when the P3a became larger. Thus, in the present study, the lack of MMN was evidently a consequence of the partly overlapping MMN and P3a responses. The response for change to cymbal elicited MMN and a large P3a in all children; response to this change differed from the responses to other timbre changes, which may be explained as follows. The spectral difference between piano and cymbal tones is extremely large. This change may have activated fresh afferent neurons which, in turn, increased the amplitudes of the MMN and P3a. Instead, the spectral (acoustic) difference was rather small in the other timbre changes. Importantly, it seems that there is a distinction between large acoustic deviance processing and contextual information processing from a very early age. In the present study, the change to cymbal could have been processed as a large physical change leading to large MMN where the overlap of P3a had small effect (the correlation was not significant between the MMN and P3a for cymbal change). In contrast, the change to cembalo and violin may have been processed as a contextual difference. This would be consistent with the proposal that the attention shift is sometimes a consequence of large physical difference and sometimes a consequence of contextual novelty (Kushnerenko et al., 2013).

### Differences between CI singers and CI non-singers

The CI singers showed signs of enhanced development of P3a through all measured change types. First, the development of timbre P3a was enhanced in the CI singers as follows. At T2 their timbre P3a became comparable to that of the NH group for changes from piano to cembalo and violin (Figure 4A). The timbre P3a of the CI singers was significantly larger and earlier at T2 than in the CI non-singers, whose timbre P3a became smaller and later over time. Second, the pitch P3a was larger and earlier in the CI singers than in CI non-singers at T1 and T2. It is even possible that the non-significant difference between the CI and NH group in pitch P3a amplitude is driven by the large P3a responses of the CI singers. Third, we found in the CI singers positive, P3a-like responses without subsequent MMN at T2 for rhythm-related, medium or large changes (gaps and changes in duration), emerging at T2. Intriguingly, these early positive responses explain the smaller MMN in the CI singers compared to the CI non-singers for duration changes at T2, and may explain why the CI group had smaller duration MMN than NH group or why P3a coincided with MMN for rhythmic changes in the CI group. Moreover, the rapid emergence (after 14–17 months) of P3a for rhythmic changes only in CI singers is consistent with the findings that P3a can emerge only after 5 days of training (Draganova et al., 2009). That they emerged to the largest changes is in turn consistent with the findings that P3a is strongly modulated by the amount of change (Wetzel et al., 2006; Koistinen et al., 2011, among others). Importantly, the enhanced production of rhythm of CI singers at T2 (Section Division of CI Group into CI Singers and CI Non-singers) supports the interpretation that the positive responses are related to better perception. Further, the positive relation of singing to P3a responses is also consistent with the findings of Putkinen et al. (2013b), showing enhanced P3a responses for gap and duration changes in two to three year old NH children with a high amount of informal musical activities including singing.

The enhanced P3a development and the emergence of new, early P3a-like responses without MMN in the CI singers may reflect more accurate perception of changes than CI non-singers, at least in the present musical context (see previous section). Moreover, due to slow-rate, predictable singing, they perhaps discriminated or began to discriminate the changes better, further increasing distractability. The simultaneous enhancement of these factors may have led to the rapid enhancement or appearance of early P3a in the CI singers.

Interestingly, the pitch MMN became larger in CI non-singers without any evidence on enlargement of the pitch P3a. The P3a enlargement should have been expected because when MMN and P3a are both elicited, P3a becomes larger with larger MMN (Draganova et al., 2009). This may reflect development of preattentive discrimination without development of more attentional discrimination in the CI non-singers. This would show similarities with normal-hearing subjects who suffer from tone-deafness, also called congenital amusia, who have near-to-normal preattentive neural processing (MMN) of musical pitch incongruities even though they have limited conscious accuracy in such a task (Peretz et al., 2009). However, another interpretation might be that the neural networks for P3a develop less accurately in the CI non-singers than in the CI singers.

The neural network for P3a is distributed across frontal, parietal and temporal (auditory) cortical regions (Takahashi et al., 2013), suggesting functional connectivity between them. Interestingly, people suffering from amusia have degraded connections between frontal and temporal regions in their right hemisphere (Loui et al., 2009). Congenital deafness can also lead to degradation in white-matter volume in the auditory cortex and thus fewer afferent and efferent fibers (Emmorey et al., 2003). Because the increase in white-matter in association cortices, important for the maturation of auditory orienting, is already strong before the age of 8–12 months in normal-hearing children (Kushnerenko et al., 2013, for a review), missing auditory input even within the first years of life may harm the neural basis of attention shift toward sounds.

Importantly, normal-hearing singers have enhanced white-matter (anatomical) connectivity between frontal and temporal cortical regions (Halwani et al., 2011), and singing-based aphasia therapy seems to lead to similar enhancement (Wan et al., 2014). Based on the present results and the former evidence, singing could help CI children toward better functioning neural networks for auditory attention. This would be in line with the faster plastic changes in auditory and frontal areas in 6 years old children participating 15 months in musical training compared to other children (Hyde et al., 2009). Importantly, the plastic changes found by Hyde et al. (2009) were accompanied with enhanced development in auditory melodic and rhythmic discrimination.

### The general importance of the present findings

The present results suggest that when the ERP-responses of CI children are compared to NH children, one should pay attention to both MMN and P3a. First, the NH peers can be proceeding toward smaller P3a when CI children are proceeding toward larger P3a. Second, the overlap of P3a with MMN can diminish the MMN. Third, the CI children who are exposed to multisensory musical activities, like singing, may differ from other CI children in the development of auditory attention shift. These aspects, if not taken into account, can affect the interpretations of the ERP responses.

The present study design cannot confirm the causality of singing in the development of attention shift. The enhanced P3a responses may be a consequence of some predispositions in CI singers which we could not find, and the CI singers may sing due to their better perceptual abilities or better neural networks for attention. However, the CI singers did not differ from CI non-singers in CI and hearing-related factors or socioeconomic or musical background (see Section Division of CI Group into CI Singers and CI Non-singers). Therefore, the present findings on P3a support the interpretation that singing enhances auditory attention and perception.

It is possible that more accurate perception of music with singing leads to enhanced enjoyment on music, which, in turn, helps children benefit more from music listening through the entire life span. Further, accurate processing of spectro-temporal changes, underlying the detection of changes in musical instrument timbre, is required for the perception of phonemes (Stevens, 1998; Patel, 2014). Perception of prosody, which is thought to assist perception and learning of spoken language, is better with good discrimination of pitch (Torppa et al., 2014) and musical rhythm (Hausen et al., 2013). It has already been suggested that improving music perception of CI listeners would enhance their speech perception (Drennan and Rubinstein, 2008). These findings are promising for the speech perception and spoken language learning of the CI singers.

Moreover, efficient attention shifts are necessary in order to process rapidly changing auditory scenes like in traffic, or in schools, day cares and other places where the attention should be directed quickly toward important sounds. It is also necessary for the further cognitive processing of incoming sounds (Friedman et al., 2001), and this enhanced attention shift toward sound changes may help the CI singers in their everyday life.

### The limitations of the present study

The present study design includes several types and magnitudes of changes in two groups of children recorded twice. This led us to conduct large amount of statistical analyses, but we corrected for multiple testing only in the *post-hoc* tests. This might lead to type 1 errors, i.e., some connections could be significant by chance. However, the results show a coherent general enhancement of P3a in CI singers. In addition, using statistical correction for reducing the responses taken into analyses would have increased the type 2 errors (missing significant responses) due to singing-related variation in the timing of the responses. For example, at T2 CI non-singers had pitch MMN in the latency window for the amplitude analyses for pitch P3a, and the CI singers had intensity, duration and gap P3a in the time window of the amplitude analyses for MMN, which inevitably reduces the significance of these responses. Therefore, we are assured that the best solution was to use relatively liberal correction procedures.

## Conclusions

Based on current MMN and P3a findings, singing may enhance perceptual and attentional functions related to music and, possibly, sounds in general. We found an interplay between development of MMN and P3a responses, hearing status, and singing of CI children. The present findings are novel especially because they are the first showing enhanced P3a responses related to singing of CI children. Even though the present study cannot show direct causality of singing to brain development and enhanced auditory processing, these findings should boost future studies on the effects of singing, especially on attention toward sounds. Singing requires no expensive musical instruments or formal instruction and is readily available for everyone. From both the economical and ethical perspectives, the present findings may be most important for the rehabilitation and quality of life of CI children. It also seems that despite of their difficulties in singing in tune (Nakata et al., 2006; Xu et al., 2009), children with CIs should be given opportunities and be encouraged to sing. Still, an important goal of future studies is to confirm the causality of the connections between singing and the advanced auditory perception and attention.

### Conflict of interest statement

The authors declare that the research was conducted in the absence of any commercial or financial relationships that could be construed as a potential conflict of interest.
