# Supplementary material for: Interplay between singing and cortical processing of music: a longitudinal study in children with cochlear implants
Source: Front Psychol. 2014 Dec 10;5:1389. doi: 10.3389/fpsyg.2014.01389 (PMC4261723; doi:10.3389/fpsyg.2014.01389)
Supplement: Supplementary file 1 [file Supplement1.DOCX]

**Supplement 1. Statistical analyses testing the differences between CI singers and CI non-singers in background**

According to analysis of variance (ANOVA), CI singing groups (CI singers and CI non-singers) did not differ significantly from each other in age. Age-controlled ANCOVA confirmed that they did not differ from each in the musical background as assessed by musical activity clusters (from a questionnaire: see Torppa et al., 2014, their Supplementary Appendix 2) measuring these aspects: Music activity at home, i.e. how many times per week parents or siblings sang or played instruments with the child or whether or not the child played instruments him/herself: How many times per week the child watched children’s music videos or DVDs (and how she/he responded to that), the child listened to music CDs, the child participated music lessons at school or daycare, the child listened to background music: The time (in months) the child had spent at musical hobbies or dancing lessons before measurements. Moreover, age-controlled ANCOVA confirmed that according to clinical records, they did not differ in hearing age, maxima (the optimal number of stimulation sites with ACE coding strategy), C levels (the loudest comfortable stimulation level on each CI channel) and dynamic ranges (inspected only in children using Nucleus devices) (see Torppa et al., 2012, their Supplementary Table 1), age at implantation, or pure tone thresholds using CI for 125 Hz, 250 Hz and mean of 500, 1000 and 2000 Hz. Chi-square statistics confirmed that CI singing groups did not differ in the attendance of musical activities outside of the home between measurements, socioeconomic background (education of father and mother, incomes of parents), device type, gender or etiology, which in the present participants was either genetic (Connexin 26) or unknown (see Table 1).
