# Supplementary material for: Interplay between singing and cortical processing of music: a longitudinal study in children with cochlear implants
Source: Front Psychol. 2014 Dec 10;5:1389. doi: 10.3389/fpsyg.2014.01389 (PMC4261723; doi:10.3389/fpsyg.2014.01389)

Supplement 2. Examples of median and average signals for the CI group.

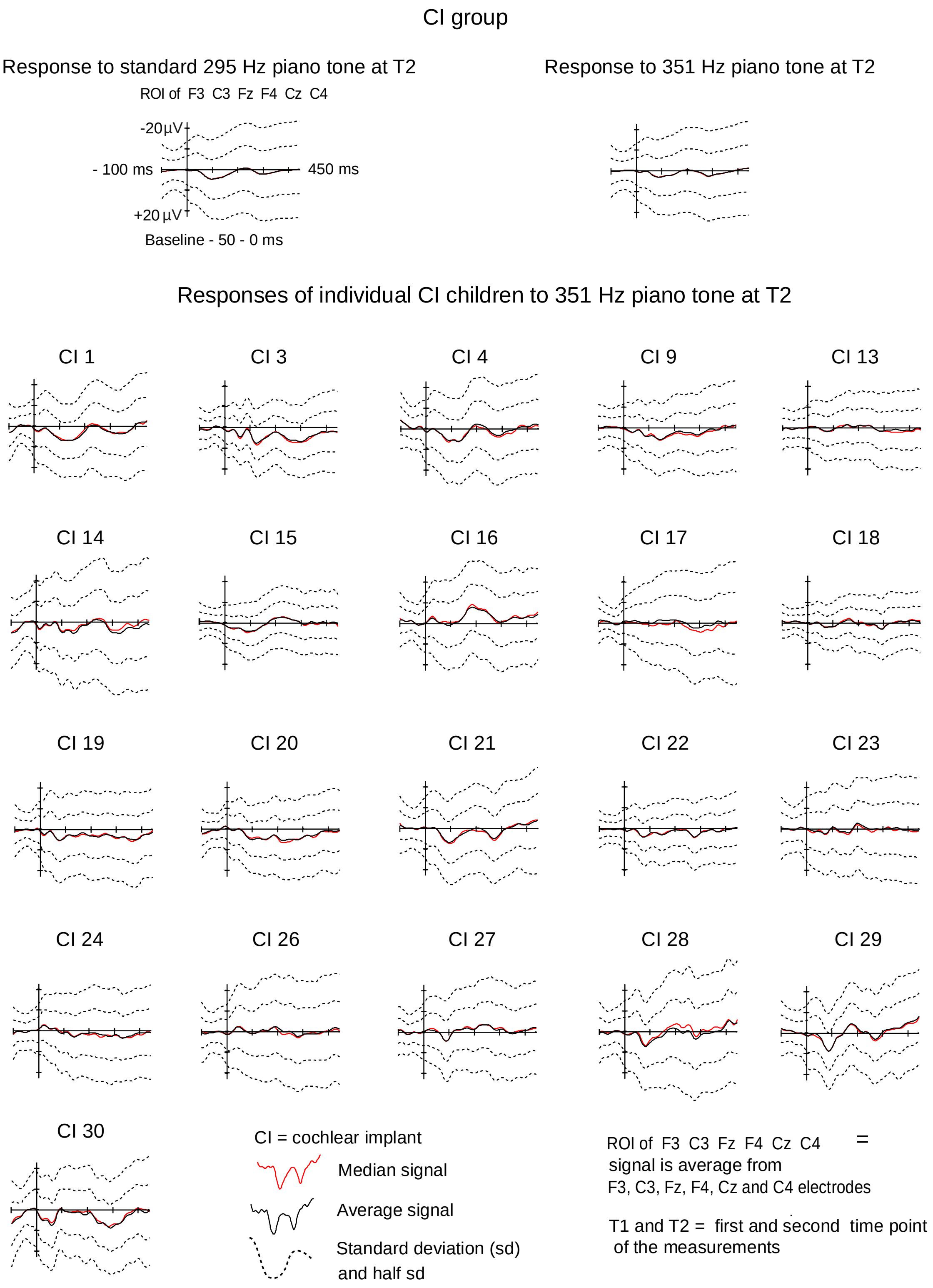

Supplement: Supplementary file 2 [file Supplement2.PDF]
