# Supplementary material for: Interplay between singing and cortical processing of music: a longitudinal study in children with cochlear implants
Source: Front Psychol. 2014 Dec 10;5:1389. doi: 10.3389/fpsyg.2014.01389 (PMC4261723; doi:10.3389/fpsyg.2014.01389)
Supplement: Supplementary file 4 [file Supplement4.PDF]

Supplement 4. The analyses and results for intensity MMN and P3a amplitudes and latencies

1. The ERP analyses

After visual inspection of the data, intensity decrement MMN was identified as the local minimum (most negative peak) of the subtraction waveform within the time window 90-350 ms after change onset, and intensity increment MMN as the local minimum within the time window 90-250 ms after change onset. Intensity increment P3a was identified as the local maximum of the subtraction waveform within the time window145-300 ms after change onset. Intensity decrement P3a was not analysed because preliminary inspection showed that it was not elicited in the entire CI group or NH group.

In the NH group, the mean amplitude of intensity increment MMN and P3a was not analysed due to different processing between CI and NH groups (see Figure 2). For example, medium and large intensity increments elicited a P3a-like response in the NH group (especially at T1) while not in the CI group. Moreover, in the CI group, for intensity 3 dB increment MMN was elicited at T1, the response changed polarity during the follow-up and only P3a was elicited at T2. By contrast, in the NH group, only negative MMN-like response was elicited at both time points.

The individual peak latencies were calculated from ROI-signals in a time window determined in relation to the change onset. Based on visual inspection of the data, the window was set at 100-400 ms for intensity MMN. The latencies for intensity 3 dB decrement P3a were not analysed.

The responses were included in further analyses for testing the hypotheses (comparing CI and NH groups as well as CI singers and CI non-singers) following similar principles as for the other change types (see section EEG Recording, Data Preprocessing and Data Analysis). However, because the response for intensity 3 dB increment changed polarity (changed from negative to positive or vice versa in the time window of MMN/P3a) during the course if this study in CI or NH group, that was not taken into further statistical analysis.

2. Hypotheses and statistical analyses for testing them

We tested two hypotheses: I) The CI children have smaller and later MMN and P3a than NH children for the changes intensity: the differences between groups become smaller over time. II) The MMN and P3a is/becomes larger and earlier in CI children who sing regularly at home compared to other CI children. The statistical analyses for testing the hypotheses were conducted similarly to the statistical analyses for the MMN and P3a responses for other measured change types (see section Statistical Analyses). In what follows, we report the findings related to the hypotheses.

3. Results

Supplementary Table 1. The intensity MMN and P3a mean amplitudes and latencies

Stimulus eliciting the response:		CI group				NH group			
		T1 µV	T2 µV	T1 ms	T2 ms	T1 µV	T2 µV	T1 ms	T2 ms
Intensity	3 dB(S)	-.43(1.99)	-.76(1.92)°	-	-	-1.20(2.55)*	-1.52(2.19)**	-	-
decrement	6 dB(M)	-.82(1.86)*	-.28(2.14)	255(83)	249(84)	-1.55(2.74)*	-1.64(2.93)*	248(78)	265(78)
MMN	9 dB(L)	-.19(2.02)	-.41(2.04)	-	-	-1.29(2.50)*	-2.46(2.65)***	-	-
Intensity	3 dB(S)	-1.26(.96)***	n	-	-	-	-	-	-
increment	6 dB(M)	-.07(1.61)	-.90(2.31)°	-	-	-	-	-	-
MMN	9 dB(L)	-.20(1.67)	-.60(1.94)	-	-	-	-	-	-
P3a	3 dB(S)	n	1.29(1.81)**	-	-	-	-	-	-

For both CI and NH groups, the mean MMN and P3a amplitude (standard deviation in parenthesis), followed by the significance of the response (˚p<.1, *p<.05, **p≤.01, ***p<.001; two-tailed t-test against zero), are given at T1 and T2. Then the mean of peak latencies (and standard deviation) of MMN and P3a are given at T1 and T2. The columns marked with gray present the amplitude and latency values included in statistical comparisons between CI group and NH group as well as between CI singers and CI non-singers. - = the mean amplitudes or individual latencies were not analyzed. n = the responses were non-existent (wrong polarity in the time window of the response). Standard was 295 Hz, 200 ms, 70 dB (for CI group)/60 dB (for NH group) piano tone. S, M, L = small, medium and large amount of change.

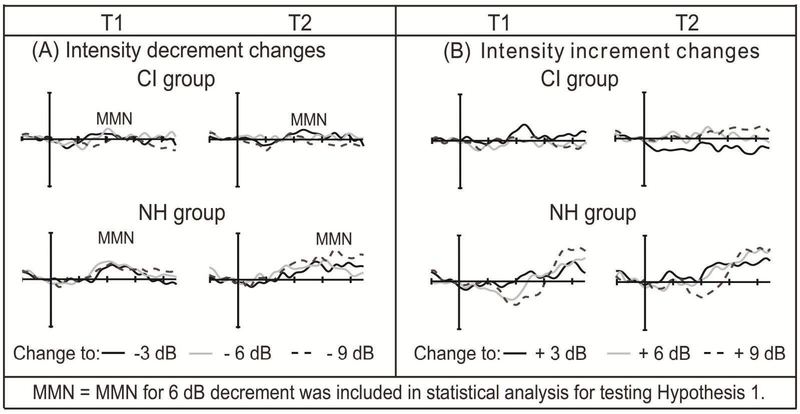

Supplementary Figure 1. The subtraction (deviant – standard) ROI waveforms averaged across F3, Fz, F4, C3, Cz and C4 electrodes for CI and NH group for (A) intensity decrement changes, and (B) intensity increment changes. They are given for both time points of the measurements (T1 and T2 on the left and right in each panel, respectively).

The statistical differences between CI and NH group in intensity 6 dB decrement MMN (the only response included in the analyses for testing the hypotheses, Supplementary Table 1) were not significant (Supplementary Figure 1 A).

Supplementary Table 2. Results (unstandardized estimates for main effects) for testing Hypothesis II

	Intensity 6 dB decrement	
	Amplitudes	
	B	*F*
CI singing group	-3.34	14.39**
Time	-1.27	1.5

CI singing group = CI singers vs. CI non-singers. For *B*: *CI singing group*: reference is the CI singers. *Time*: reference is the T2.
˚p < .1, *p < .05, **p ≤ .01, ***p ≤ .001, two-tailed t-test against zero.

For testing Hypothesis II, the main effect of *CI singing group* was significant. The CI singers had significantly smaller intensity 6 dB decrement MMN than the CI non-singers (Supplementary Table 2). However, in the CI singers, the difference wave was already positive in the time line of MMN at T1 and T2 (Supplementary Figures 2 A, 3 A and 4).

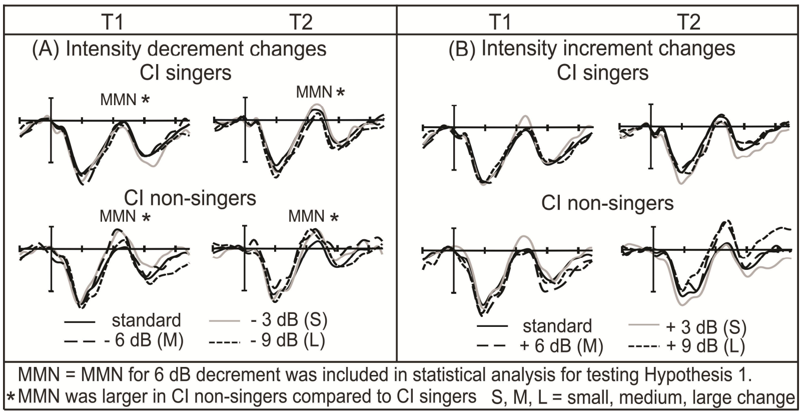

Supplementary Figure 2. The ERP ROI waveforms averaged across F3, Fz, F4, C3, Cz and C4 electrodes for CI singers and CI non-singers for standard tones and for **(A)** intensity decrement changes, and **(B**) intensity increment changes. They are given for both time points of the measurements (T1 and T2 on the left and right in each panel, respectively).

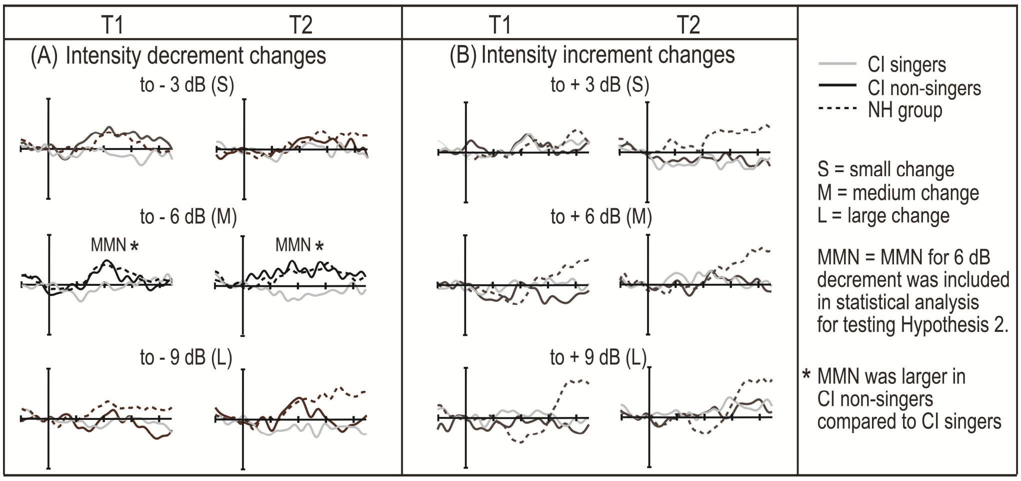

Supplementary Figure 3. The subtraction (deviant – standard) ROI waveforms averaged across F3, Fz, F4, C3, Cz and C4 electrodes for NH group, CI singers and CI non-singers for **(A)** intensity decrement changes, and **(B**) intensity increment changes. They are given for both time points of the measurements (T1 and T2 on the left and right in each panel, respectively).

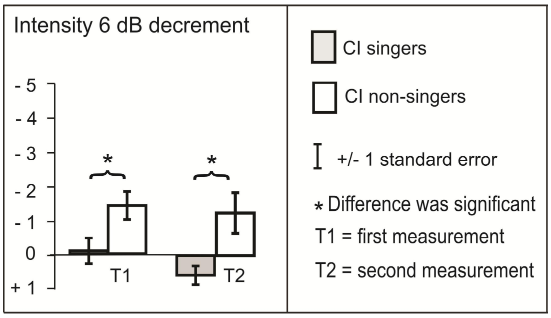

Supplementary Figure 4. The illustration of the significant difference between CI singers and CI non-singers on 6 dB intensity decrement.
