# Supplementary material for: Interplay between singing and cortical processing of music: a longitudinal study in children with cochlear implants
Source: Front Psychol. 2014 Dec 10;5:1389. doi: 10.3389/fpsyg.2014.01389 (PMC4261723; doi:10.3389/fpsyg.2014.01389)
Supplement: Supplementary file 5 [file Supplement5.PDF]

Supplement 5. The ERP ROI waveforms averaged across F3, Fz, F4, C3, Cz and C4 electrodes for CI and NH group for standard tones and for (**A**) timbre changes, (**B**) pitch changes (**C**) intensity decrement changes, (**D**) intensity increment changes, **(E)** gap changes and **(F)** duration changes. The ERP waveforms are given for both time points of the measurements (T1 and T2).

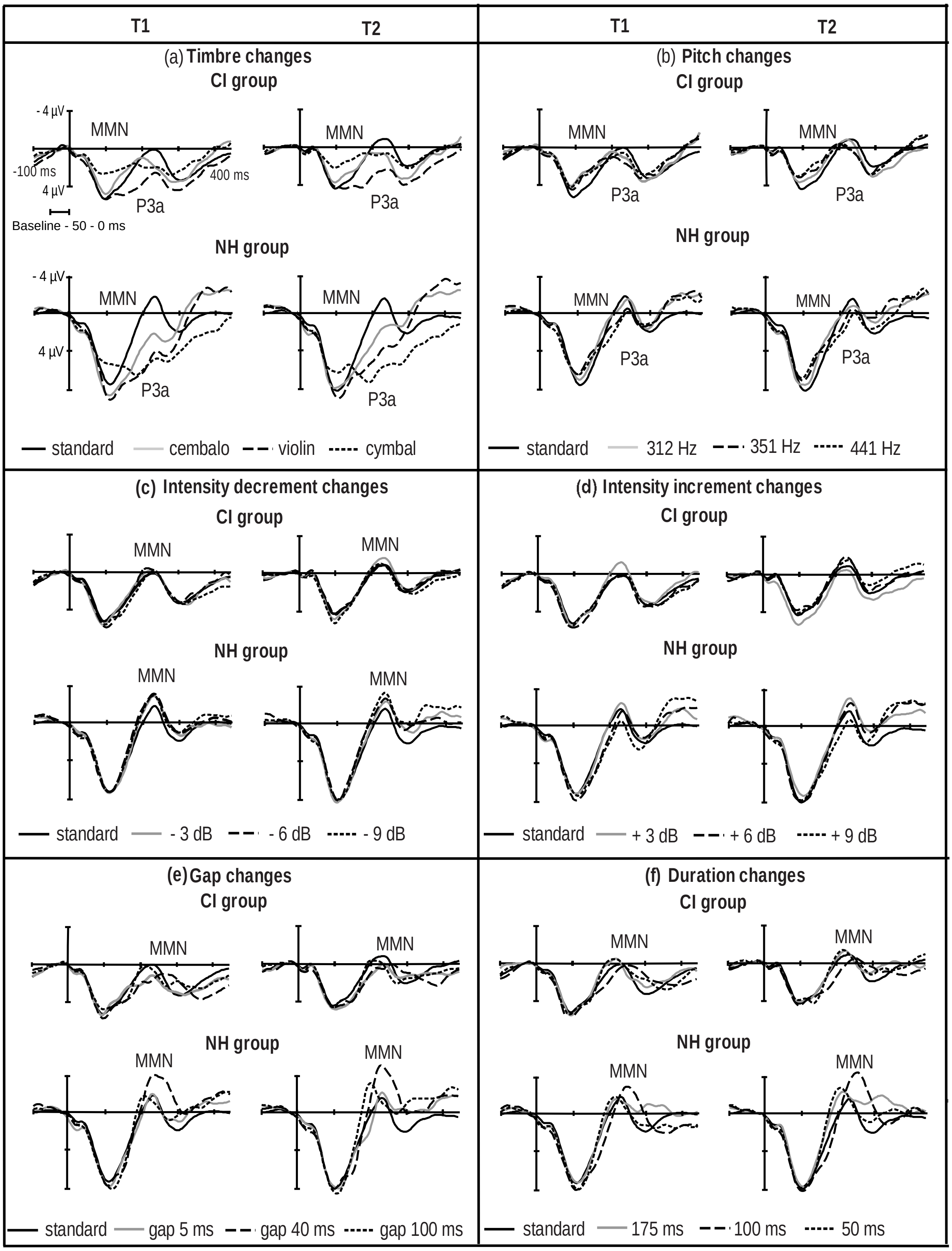

"MMN" and "P3a" indicates that response for one or more amount of changes was included in statistical analysis for testing Hypothesis 1.
